# Monitoring Environmental Glyphosate in Northeastern Romania and Its Cytotoxic Impact on Human Fibroblasts

**DOI:** 10.3390/jox16020061

**Published:** 2026-04-02

**Authors:** Ioana-Cezara Caba, Raluca Stefănescu, Alexandra-Andreea Botnaru, Ionela Daniela Morariu, Liliana Vereștiuc, Florina-Daniela Cojocaru, Bogdan Caba, Oana Cioancă, Alexandra Jităreanu, Luminița Agoroaei

**Affiliations:** 1 Faculty of Pharmacy, Grigore T. Popa University of Medicine and Pharmacy, 700115 Iasi, Romania; ioana-cezara.caba@umfiasi.ro (I.-C.C.); ionela.morariu@umfiasi.ro (I.D.M.); oana.cioanca@umfiasi.ro (O.C.); jitareanu.alexandra@umfiasi.ro (A.J.); luminita.agoroaei@umfiasi.ro (L.A.); 2 Advanced Research and Development Center for Experimental Medicine “Prof. Ostin C. Mungiu”—CEMEX, Grigore T. Popa University of Medicine and Pharmacy, 16 Universitătii Street, 700115 Iasi, Romania; raluca.stefanescu@umfiasi.ro; 3 Faculty of Medical Bioengineering, Grigore T. Popa University of Medicine and Pharmacy, 700020 Iasi, Romania; liliana.verestiuc@bioinginerie.ro (L.V.); florina.cojocaru@umfiasi.ro (F.-D.C.); bogdan-caba@umfiasi.ro (B.C.)

**Keywords:** glyphosate, environmental contamination, in vitro cytotoxicity, MTT assay, human gingival fibroblasts

## Abstract

Glyphosate is the most widely used pesticide globally, raising concerns about its environmental persistence and biological impacts. Therefore, monitoring pesticide use is essential for assessing agricultural practices and the risks to human health associated with chemical use. This research examined glyphosate contamination in water (40 samples) and soil (28 samples) from northeastern Romania, an important agricultural region. Glyphosate concentrations in environmental water and soil samples were quantified using a spectrophotometric method based on ninhydrin derivatization, with good linearity over the concentration range 1–30 µg/mL (R^2^ = 0.9981). Glyphosate was detected at concentrations above the LOQ in one water sample. Also, the study proposes a UHPLC-MS/MS method for the confirmation of glyphosate presence in the analyzed sample. Additionally, this study contributes to the characterization of the toxicity profiles of glyphosate and a commercial glyphosate-based formulation (Roundup^®^) in primary human gingival fibroblast (hGF) cell lines. The commercial product Roundup, containing glyphosate, exhibited cytotoxicity similar to that of the active compound at low and intermediate doses; a significant cytotoxic effect was observed at a maximum concentration of 1 mM, with prolonged exposure. These findings demonstrate minimal cytotoxicity under the examined conditions and underscore the need for dose- and time-dependent assessments to evaluate the biological impact of herbicide formulations.

## 1. Introduction

The widespread application of pesticides has contaminated food, feed, water, air, soil, and other resources, presenting significant threats to food safety and public health. Approximately 0.1% of the applied pesticide accomplishes its intended target, while the residual quantity becomes a contaminant in the soil and environment, consequently endangering future food sources [1,2].

Glyphosate [N-phosphonomethyl-glycine] is a broad-spectrum, post-emergent, systemic herbicide utilized for weed management, noted for its high efficacy, ease of application, and cost-effectiveness, which is applicable to various crops such as soybeans, wheat, cotton, and maize. It was formulated in the 1970s by the agricultural company Monsanto and launched commercially as Roundup^®^ [3]. Glyphosate can enter aquatic environments through soil leaching, runoff, and, occasionally, through the direct application of certain permitted formulations. Furthermore, glyphosate can affect nontarget plants through several mechanisms, including spray application, translocation from treated plant tissues, and decomposition of weed remnants [4,5].

Despite substantial evidence of human exposure, the European Food Safety Authority, the Environmental Protection Agency, and the U.S. National Cancer Institute have declared glyphosate acceptable for human consumption. This conclusion is based on toxicity data and the absence of glyphosate-inhibited enzyme activity in humans. Conversely, increasing research indicates that glyphosate and glyphosate-based herbicides exhibit cytotoxic, genotoxic, and endocrine-disrupting properties, possibly affecting human health [6,7,8]. Moreover, glyphosate is a significant environmental factor in the development of disorders linked to gut microbiota dysbiosis, such as inflammatory bowel disease [9,10]. These controversies urge environmental and agricultural researchers to focus on this pesticide.

The EU Directive 2020/2184 on the quality of water intended for human consumption sets a limit of 0.1 µg/L for individual pesticides in drinking water. This applies to all active substances in pesticides, including glyphosate. The Directive also sets a limit of 0.5 µg/L for the amount of all pesticides in water. The EU Directive 2024/1785 on soil monitoring provides only normal values, an alert threshold, and an intervention threshold.

The primary source of chronic exposure to glyphosate for humans and animals may be food commodities. Residues of glyphosate and its commercial derivatives have been detected in both animal and plant food items. Plants exhibit greater bioconcentration and absorption capacities than herbivores. This arises due to the high solubility of glyphosate in water, facilitating its excretion via the kidneys [11]. Researchers concluded that pesticide co-formulants, often considered inert, can exert endocrine-disrupting effects at concentrations far lower than the active ingredient itself, underscoring the need to assess the toxicity of commercial formulations rather than active compounds alone [12,13].

Children are a subpopulation among the exposed populations that have particular exposure characteristics and heightened susceptibility to environmental toxins. Children may also differ from adults in detoxification levels, DNA repair mechanisms, and cell proliferation [14]. Beyond environmental contamination, pesticide exposure in northeastern Romania has also been associated with clinical intoxication cases, including pediatric patients, underscoring the real-world health relevance of monitoring pesticide residues in the region and the need for integrated environmental and medical surveillance approaches [15].

In the last two decades the identification and quantification of glyphosate was carried out using liquid-chromatography coupled with mass spectrometry in various matrices such as biological samples from human individuals (plasma or serum, milk, urine), water, soil, and food matrices (plants, wheat flour, honey). Many of the methods developed worldwide include a derivatization step using 9-fluorenylmethylchloroformate (Fmoc) [16]. Recently, derivatization using 6-aminoquinolyl-N-hydroxysuccinimidyl carbamate (AQC) was introduced [17,18]. Rapid analysis methods which do not employ derivatization were also used for the investigation of glyphosate in different matrices: the use of the ion-pairing agent heptafluorobutyric acid [19,20,21] and identification of chromatography columns able to separate this chemical compound (Hypercarb Luna-NH_2_) [22,23]).

Glyphosate has been detected in a broad spectrum of environmental and biological matrices across continents, reflecting its pervasive use in agriculture, forestry and urban settings. Monitoring initiatives indicate that glyphosate and its primary degradation product are frequently detected in these compartments, exhibiting varying concentration profiles dependent on land use and regional hydrology [24,25]. Glyphosate exhibits significant environmental persistence, with reported soil half-lives ranging from weeks to over a year, significantly influenced by soil type, microbial activity, and climatic conditions. A total of 21% of soil samples analysed in the European Union revealed the presence of glyphosate and its metabolite, aminomethylphosphonic acid (AMPA), with the highest concentrations reaching 2 mg/kg [26]. This widespread contamination poses ecological risks, as glyphosate residues can negatively affect aquatic organisms, alter microbial communities, and contribute to eutrophication through the release of phosphorus [27]. Human biomonitoring consistently reveals urinary glyphosate and its primary metabolite AMPA, with detection rates ranging from 20% in Irish adults to 86% among Chinese production workers, and median concentrations varying from 0.09 µg L^−1^ in the German general population to 0.292 mg L^−1^ in occupationally exposed Chinese workers; occupational studies further show strong correlations between air-borne levels and urinary excretion [28,29,30]. Research worldwide indicates that glyphosate is predominantly found in grain-based foods, with a detection rate of approximately 42% across 7.955 Canadian retail samples. Conversely, fresh fruits, vegetables, and infant meals typically exhibit non-detectable or trace residues, as evidenced by Italian baby-food assessments showing no glyphosate. Regional analyses indicate elevated residues in specific root crops, such as cassava in Nigeria at 0.3 mg kg^−1^ compared to maize at 0.07 mg kg^−1^, highlighting that matrix type and farming techniques influence the global variability of glyphosate levels in food [31,32,33].

Glyphosate exhibits cytotoxic and genotoxic effects in cell-based assays at millimolar concentrations, while chronic in vivo studies in rodents have demonstrated multi-organ toxicity following prolonged exposure, including liver damage (elevated AST/ALT and necrosis), renal tubular injury, endocrine disruption of the hypothalamic–pituitary–gonadal axis, cardiac arrhythmias, pulmonary inflammation, and neurobehavioral deficits in offspring. Notably, while pure glyphosate generally shows limited cytotoxicity in vitro, glyphosate-based herbicide formulations, particularly those containing surfactants such as POEA and ethoxylates, exhibit significantly higher toxicity, impairing hepatocyte, renal, and Sertoli cell viability, disrupting mitochondrial function, altering cardiomyocyte contractility, and inducing genotoxic effects [26,34].

Given concerns about human health, environmental impacts, and the potential for resistance, several governments have prohibited the use of glyphosate. The US and the EU have not enacted a total ban; nevertheless, certain limits have been instituted, and negotiations over a possible phase-out are in progress [35].

The advancement of analytical techniques for glyphosate assessment has risen in recent years, with several quantification approaches being suggested [36,37,38,39,40]. Nonetheless, most analytical techniques require expensive, advanced equipment, rendering them economically inaccessible to most laboratories. In certain situations, it is preferable to use a simpler, quicker method that incurs lower analytical costs than chromatographic techniques, despite its reduced sensitivity.

Despite the widespread use of glyphosate-based herbicides, data on its presence in the environment in Eastern European regions remain limited. In the North-Eastern region of Romania, to our knowledge, no monitoring studies have been conducted, despite its intensive agricultural practices and potential exposure risks.

Furthermore, although the toxicity of glyphosate has been investigated in various cell models, there is a lack of data on its effects on human gingival fibroblasts, with oral exposure being of particular concern (glyphosate residues can be ingested through water and plant foods).

The novelty of the present study lies in the combined approach, integrating environmental monitoring of glyphosate in a previously uncharacterized region and in vitro toxicological evaluation in a biologically relevant human cell model associated with oral exposure.

Considering these environmental and toxicological issues, ongoing surveillance of glyphosate residues in environmental samples along with evaluations of biological impacts are essential for understanding the potential hazards associated with its widespread use. This study aims to quantitatively assess glyphosate levels in water and soil samples by a spectrophotometric method and to examine the cytotoxic effects of glyphosate and glyphosate-based pesticide formulations on the hGF cell line.

## 2. Materials and Methods

The Ethics Committee of Grigore T. Popa University of Medicine and Pharmacy approved the study design (No. 90/08.06.2021). Microsoft Excel and SPSS Statistics (version 26.0) were used for data analysis.

### 2.1. Study Area and Sampling Sites

**Figure 1:**
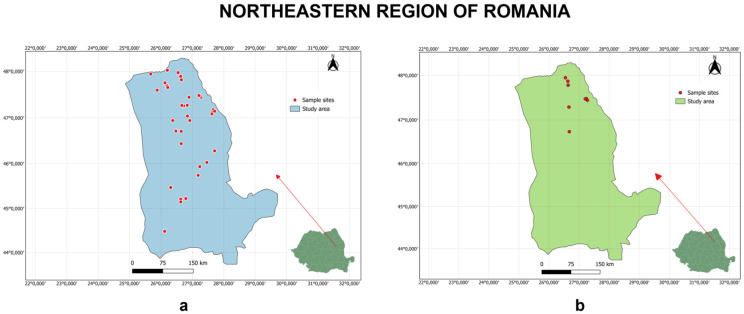
Geographical location of water (**a**) and soil (**b**) sampling sites in northeastern Romania (Created with QGIS).

Environmental water and soil samples were collected from the northeastern region of Romania. The geographical distribution of the sampling sites of water (Figure 1a) and soil (Figure 1b) is illustrated in Figure 1.

The combination of soil vulnerability and increased rainfall erosivity in north-eastern Romania enhances the risk of pesticide transport through runoff and leaching processes [41]. Consequently, systematic monitoring of pesticide residues is essential to prevent soil degradation, protect water resources, and ensure sustainable agricultural productivity.

The selected sites were located either in the proximity of agricultural areas or near residential households with private gardens used for crop cultivation. The surrounding land was characterized by typical regional crops, including wheat, maize, sunflower, and, depending on the year, rapeseed, reflecting the crop-rotation practices commonly applied in the area. Based on surrounding land use and proximity to active fields, the sites were broadly classified as moderate- or high-intensity agricultural areas. Only a limited number of areas in the region are managed under organic farming practices.

A total of 28 soil samples were collected from agricultural areas in north-eastern Romania, during the period March–April. Soil samples were collected from agricultural areas in north-eastern Romania. Sampling sites were selected based on their agricultural relevance and potential exposure to glyphosate application. Samples were collected randomly from the topsoil layer (0–20 cm), which represents the most active zone for pesticide accumulation. At each sampling location, three sub-samples were collected and subsequently pooled to obtain a representative composite sample. Each composite sample weighed approximately 500 g. The collected samples were placed in clean, labeled polyethylene bags and transported to the laboratory on the same day.

Water samples were collected from 40 locations in north-eastern Romania, including both surface water sources and groundwater (wells), during the period March–April. Sampling sites were selected based on their proximity to agricultural land and the likelihood of glyphosate contamination through runoff. At each site, water samples were collected manually using clean, pre-labeled polyethylene bottles. For surface water, samples were collected just below the water surface to avoid debris and surface films. For groundwater, samples were collected directly from wells after allowing the water to run briefly to obtain a representative sample. All samples were transported to the laboratory and stored at −20 °C until analysis.

### 2.2. Chemicals, Reagents, and Equipment

Analytical-grade reagents were used throughout the study, including acetic acid (p.a.), sodium acetate, sodium molybdate (Na_2_MoO_4_), and ninhydrin. The glyphosate standard was purchased from Sigma-Aldrich (Darmstadt, Germany). Ultrapure Type I water (conductivity 0.45–0.46 µS/cm) was used for all solution preparations. Spectrophotometric measurements were performed using a NanoDrop One spectrophotometer (Thermo Fisher Scientific, Waltham, MA, USA) in cuvette mode.

Primary hGFs were obtained from Cell Lines Service (CLS, Eppelheim, Germany; code 300703; lot 300703-1541SF2) and used at passage 7. For the in vitro cytotoxicity assays, DMEM F-12 HAM culture medium supplemented with L-glutamate, sodium pyruvate (0.55 g/L), and glucose (3.15 g/L), trypsin from porcine pancreas (CAS 9002-07-7), penicillin/streptomycin/neomycin mixture (P/S/N), fetal bovine serum (FBS), MTT reagent (CAS 298-93-1), Hank’s Balanced Salt Solution (HBSS, without CaCl_2_ and MgSO_4_), and DMSO (dimethyl sulfoxide) were used. All reagents were purchased from Sigma-Aldrich (Germany). Roundup^®^ is an herbicide containing 360 g/L glyphosate and was purchased from Bayer Romania (Bucharest, Romania).

A high-performance liquid chromatography coupled with high-resolution mass spectrometry (UHPLC–UHRMS/MS) using a Thermo Scientific Orbitrap 480 system manufactured by Thermo Scientific (Germering, Germany), equipped with a heated electrospray ionisation (HESI) source in negative mode was developed for the identification of glyphosate. Chromatographic separation was performed on a Hypercarb (porous graphitic carbon) column (5 µm, 100 mm × 4.6 mm), Country of Origin USA, suitable for highly polar compounds such as glyphosate.

Acquisition was performed in Full MS and MS/MS (HCD) modes, with monitoring of the precursor ion corresponding to glyphosate [M-H]^−^ at *m*/*z* 168.0072 in an appropriate mass range. Fragmentation of the precursor ion was performed at a collision energy of 40 HCD, for structural confirmation, and the data were processed using standard UHRMS criteria: exact mass, retention time, and characteristic fragmentation spectrum.

### 2.3. Quantitative Analysis of Glyphosate by Spectrophotometric Method Coupled with Confirmatory Determination by UHPLC-MS/MS

The spectrophotometric quantification of glyphosate employing ninhydrin derivatization in conjunction with sodium molybdate was adapted from the method described by Rodríguez et al. [42]. The method was applied to quantify glyphosate in water and soil samples.

#### 2.3.1. Sample Collection and Preparation

Water samples were directly analyzed without intermediate processing steps. Glyphosate quantification in soil samples involved three steps: (I) soil sample preparation; (II) ninhydrin derivatization; (III) spectrophotometric quantification. To mitigate potential interference from variable humidity content, 500 mg of each soil sample was incubated at 85 °C and 400 rpm for 30 min in a Thermomixer before analysis. Before spectrophotometric analysis, 200 mg of dried soil was treated with 0.5 mL of type I water, stirred for 30 min at room temperature and 1400 rpm in a thermomixer, then filtered through 0.22 µm filters. A total of 300 µL was treated with the same amount of sodium molybdate and ninhydrin and processed according to the method described above.

#### 2.3.2. Preparation of Stock Solutions and Reagents

A standard solution of glyphosate (5 mg/mL) was prepared by dissolving pure glyphosate powder in Type I water and vortexing until fully dissolved. A glyphosate stock solution (100 µg/mL) was prepared by diluting the standard solution with Type I water. A 2.5% (*w*/*v*) ninhydrin solution was obtained by dissolving 1.25 g of ninhydrin in 50 mL of a 3:1 (*v*/*v*) combination of Type I water and acetate buffer (0.4 M, pH 5.5). A 5% (*w*/*v*) sodium molybdate solution was prepared by dissolving 2.50 g of anhydrous sodium molybdate in 50 mL of Type I water.

#### 2.3.3. Derivatization Procedure and Spectrophotometric Analysis

Derivatization was performed at a final volume of 900 µL by combining the diluted sample solution, ninhydrin working solution, and 5% sodium molybdate in a 1:1:1 (*v*/*v*/*v*) ratio. The mixture was incubated at 85 °C for 30 min at 300 rpm in a Thermomixer.

Upon reaching room temperature, the solution was placed in a cuvette, and absorbance was measured at 567 nm. Rotation speeds above 300 rpm were avoided, as such speeds detrimentally affected the intensity of the violet-coloured complex and compromised linearity. Calibration curves were established by spiking ultrapure water with standard glyphosate, and linearity, limits of detection (LOD), limits of quantification (LOQ), and accuracy were assessed.

#### 2.3.4. Confirmatory UHPLC-MS/MS Method for Glyphosate Qualitative Analysis

The method used 0.1% formic acid in water (A) and 0.1% formic acid in methanol (B) in a ramp from 95:5 to 10:90 in 15 min, partial loop injection of 10 μL, a debit flow of 750 μL, Hypercarb column (100 mm × 4.6 mm, 5 μm) at 40 °C, and the instrument settings were electrospray ionization (H-ESI), scan type SRM, negative ion mode, mass range *m*/*z* from 100 to 400 at 5 Hz frequency, negative voltage of 3.0 kV and an ionization temperature of 375 °C.

Glyphosate was detected as the deprotonated ion [M-H]^−^, with experimental *m*/*z* values ranging from 168.0072 to 168.0084 and a mass error of less than 5 ppm relative to the theoretical value.

### 2.4. In Vitro Cytotoxicity Assessment

Cells were plated in 96-well plates at a density of 2 × 10^3^ cells/well and incubated for 24 h at 37 °C, 5% CO_2_, and 95% relative humidity in full DMEM F12-HAM medium supplemented with 10% FBS and 1% P/S/N.

Glyphosate and the commercial formulation Roundup^®^ (360 g/L glyphosate) were solubilized in complete culture medium and agitated at 100 rpm and 37 °C for 24 h to obtain 1 mM stock solutions. For Roundup^®^, the stock solution was prepared reported to the active ingredient, 1 mM Roundup means 1 mM of pure glyphosate. Final working solutions were obtained by sterile filtration through 0.22 µm filters. Moreover, it is important to mention that the commercial formulation contains glyphosate in form of isopropylamine salt and not pure glyphosate as the standard does.

The MTT assay was conducted with five concentrations: 1 μM, 10 μM, 250 μM, 500 μM, and 1 mM (3 technical replicates, 1 biological replicate). The culture medium was replaced with the MTT working solution (5% MTT in serum-free medium), and the cells were incubated for 2 h and 30 min at 37 °C. MTT reduction results in the formation of insoluble formazan crystals in metabolically active cells. Formazan was solubilized in DMSO, and absorbance was measured at 570 nm using a Tecan Sunrise plate reader. The assay was performed at 3, 6, and 7 days post-exposure, in triplicate. Cell viability (V) was determined as follows:

$$V\;=\;\frac{\mathrm{Absorbance}\;\mathrm{of}\;\mathrm{Treated}\;\mathrm{Cells}}{\mathrm{Absorbance}\;\mathrm{of}\;\mathrm{Control}\;\mathrm{Cells}}\;\times \;100$$

## 3. Results

### 3.1. Quantitative Determination of Glyphosate

#### 3.1.1. Linearity

The linearity of the spectrophotometric method for glyphosate determination was assessed using five independent sets of working solutions prepared from a glyphosate stock solution at concentrations of 1–30 µg/mL. Each concentration level was analyzed under identical experimental conditions as described in the Section 2.

**Figure 2:**
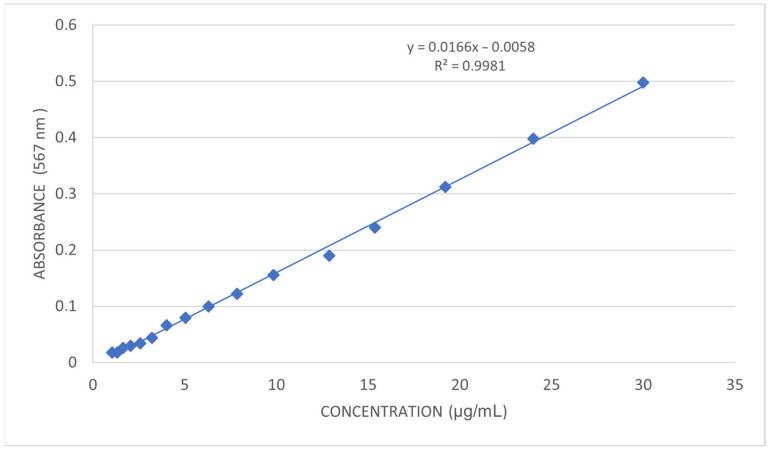
Calibration curve for the spectrophotometric determination of glyphosate in the concentration range of 1–30 µg/mL.

The experimental absorbance values and intra-assay variability are presented in Table 1. A calibration curve was constructed by plotting the mean absorbance values against glyphosate concentration. The resulting calibration curve is shown in Figure 2. A linear relationship was observed across the investigated concentration range, with a coefficient of determination (R^2^) of 0.9981, indicating excellent linearity.

The regression parameters are summarized in Table 2. The calibration equation obtained was:

Absorbance = 0.0166 × C (µg/mL) − 0.0058

This equation was subsequently used to quantify glyphosate in environmental samples.

The calibration curve showed excellent linearity in the range 1–30 µg/mL (R^2^ = 0.9981). The regression equation Abs = 0.0166 × C − 0.0058 was used to quantify glyphosate in environmental samples.

#### 3.1.2. Limit of Detection and Quantification

**Figure 3:**
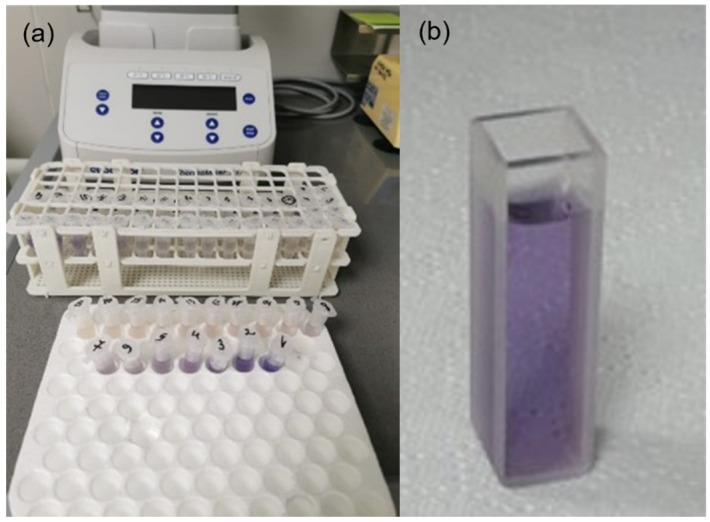
Ninhydrin derivatization of glyphosate: (**a**) reaction tubes after incubation; (**b**) violet–coloured glyphosate–ninhydrin complex formed after derivatization.

The LOQ was defined operationally as the lowest calibration level within the linear range that yielded a reproducible absorbance signal, specifically 4.026 µg/mL. Concentrations below this level were reported as below LOQ. LOD is 2.57 µg/mL. The formation of the characteristic violet-colored complex resulting from the reaction between glyphosate and ninhydrin in the presence of sodium molybdate is illustrated in Figure 3.

#### 3.1.3. Precision of the Analytical Method

The precision of the method was evaluated by assessing intra- and inter-assay variability. Intra-assay precision was assessed using replicate measurements at each concentration level within the calibration range. Inter-assay precision was evaluated by analyzing six independently prepared glyphosate stock solutions followed by serial dilutions covering the same concentration range (1–30 µg/mL). The corresponding mean values, standard deviations, and coefficients of variation (CV%) are presented in Table 3.

Overall, the method demonstrated acceptable repeatability and reproducibility, with lower variability observed at medium and high concentration levels, confirming the robustness of the analytical procedure.

#### 3.1.4. Glyphosate Concentrations in Water Samples

Glyphosate concentrations in environmental water samples were determined using a freshly prepared calibration curve on the day of analysis. The analytical results are summarized in Table 4. In addition, five commercially bottled drinking water samples were also analyzed, and the corresponding results are presented in Table 5.

#### 3.1.5. Glyphosate Quantification in Soil Samples

**Figure 4:**
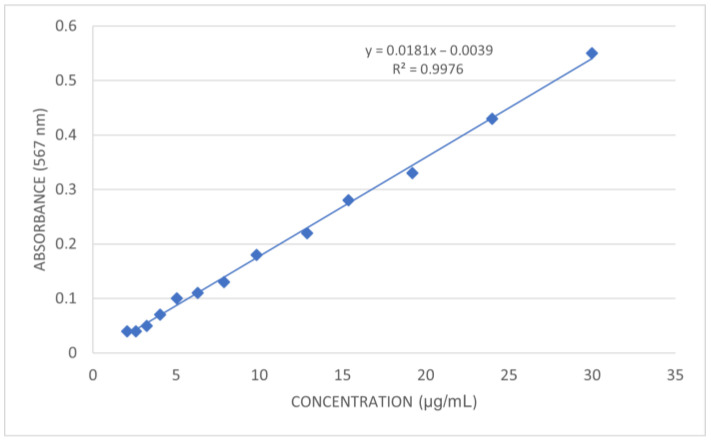
Calibration curve—spectrophotometric quantification of glyphosate in soil samples.

Glyphosate concentrations in soil samples were quantified using the same spectrophotometric method and freshly prepared calibration solutions (Figure 4). The results are presented in Table 6.

#### 3.1.6. pH of Soil Extraction Solutions

**Figure 5:**
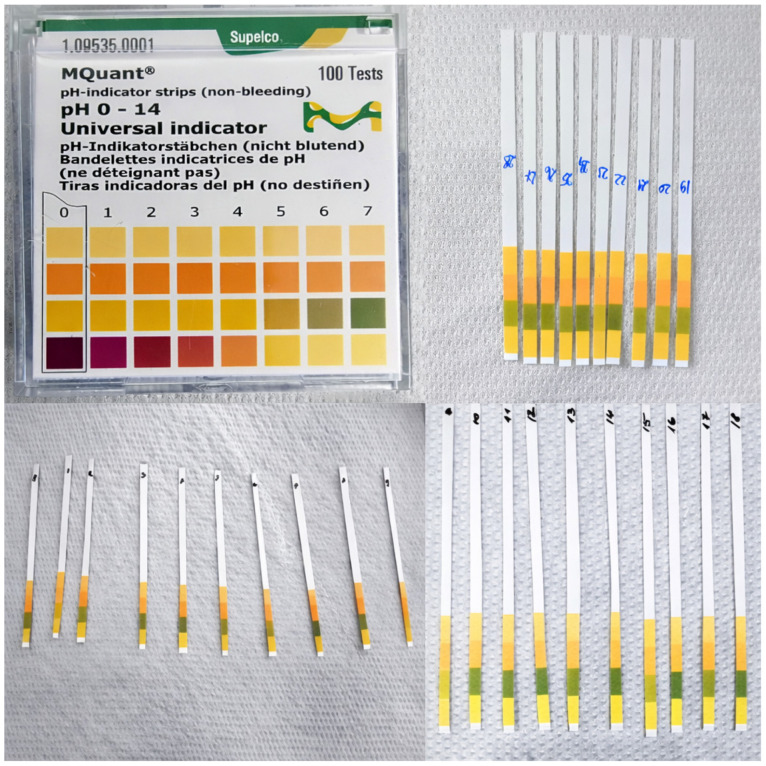
Representative pH strip readings of soil extraction solutions used for glyphosate analysis.

The pH of the soil extraction solutions was measured to assess potential influences on the glyphosate extraction method and derivatization efficiency. The pH band readings are shown in Figure 5. In general, the extraction solutions showed comparable pH values, indicating favorable conditions for both the ninhydrin-based derivatization protocol and the spectrophotometric determination.

#### 3.1.7. UHPLC-MS/MS Method for Glyphosate Confirmation

The highly polar character and low retention on conventional C18 columns necessitated the use of a Hypercarb column, which provided adequate chromatographic retention and efficient separation, facilitating the detection and confirmation of the analyte.

The analyte exhibited a well-defined chromatographic peak at 3.15–3.34 min.

**Figure 6:**
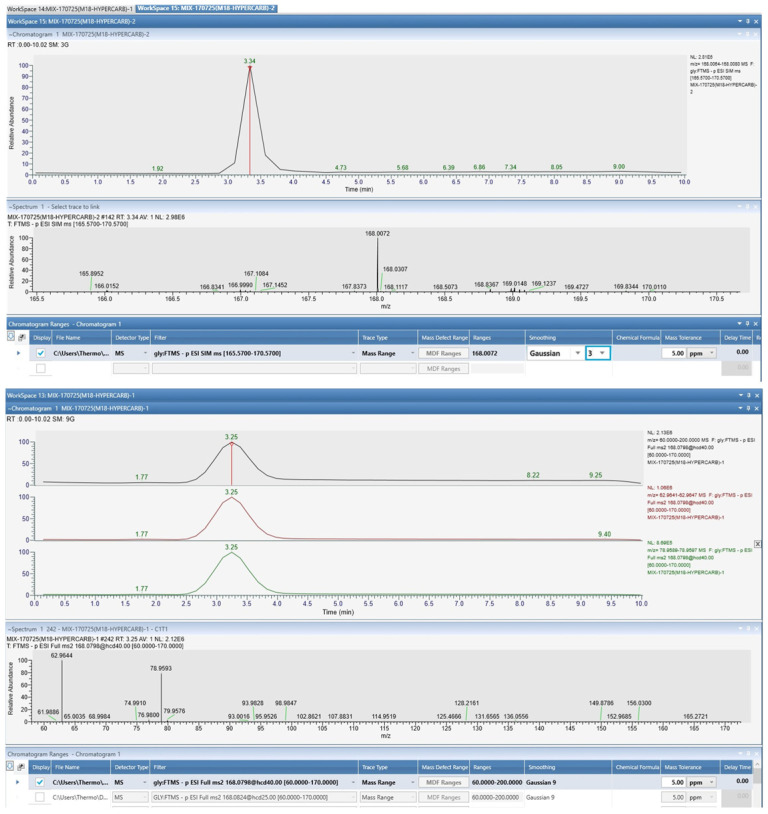
Qualitative analysis by UHPLC-MS/MS for glyphosate confirmation (precursor *m*/*z* 168.0072 and fragments *m*/*z* 63 and 79 at the retention time of 3.25).

The MS/MS spectrum of the glyphosate showed fragments characteristic of glyphosate (*m*/*z* 78.96 and 62.96). Co-elution of the precursor ion with the fragment ions, as well as the agreement of the exact mass and retention time, confirms the presence of glyphosate (Figure 6).

### 3.2. In Vitro Cytotoxicity Results

As mentioned before, for this assay, passage 7 of a primary hGF was used. HGFs are primary cells isolated from gingival connective tissue and represent a significant in vitro model for the study of oral cavity response to inflammatory stimuli [43].

According to Alehashem et al. [44], more than 100 pesticides (including glyphosate) were studied on ten human-derived cell types, but not on fibroblast cell lines. More recently, Batista et al. [45] published a study where glyphosate-based pesticides were tested in contact with human fibroblast from skin; however, to our knowledge, hGFs were never used. Studying the impact of glyphosate and Roundup^®^ on cells isolated from the oral cavity is important for better understanding their potential impact on human health, since the exposure to these pesticides in the oral cavity was related with caries, periodontal diseases, odontogenic infections and even oral cancer [46].

**Figure 7:**
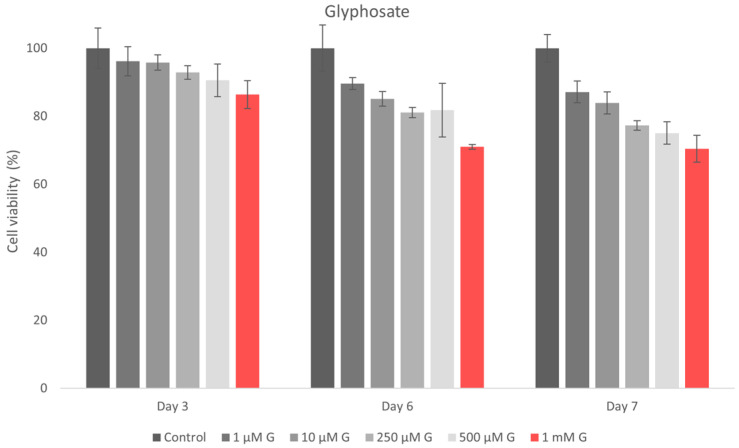
Effects of glyphosate on the viability of hGF.

The in vitro cytotoxicity of glyphosate (G) and the commercial glyphosate-based product Roundup^®^ (360 g/L) was assessed using the MTT test on hGF. Cell viability was evaluated after 3, 6, and 7 days of exposure to increasing doses from 1 μM to 1 mM. Concentration in this range were also studied by Arrigo et al. [47] on cardiomyoblasts, since higher doses were found to cause an immediate acute cytotoxicity. All experiments were conducted in triplicate, and the data are shown as mean ± standard deviation (SD) (Figure 7, Table 7).

**Figure 8:**
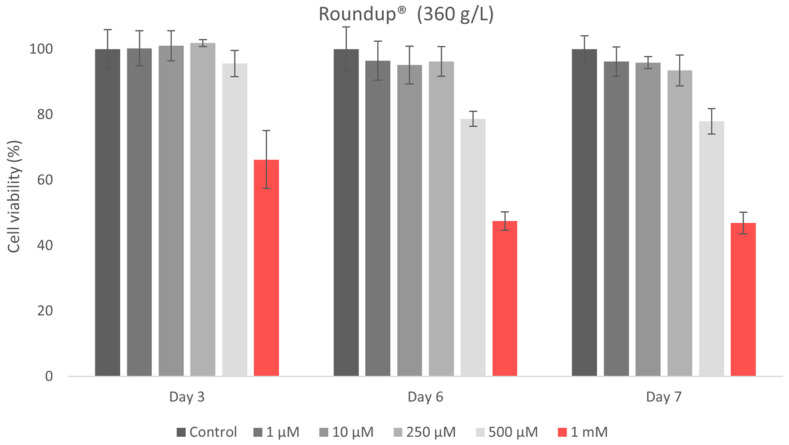
Cell viability of hGF after exposure to Roundup^®^ (360 g/L)—commercial glyphosate formulation.

Conversely, the commercial product Roundup^®^ demonstrated a significantly greater cytotoxic effect. At the maximum tested concentration (1 mM) after 3 days of exposure a mild cytotoxicity (66.23%) was observed, escalating to moderate cytotoxicity after 6 and 7 days, as indicated by the MTT findings (Table 7, Figure 8). Cell viability values for all other tested concentrations (250 µM, 10 µM, and 1 µM) exceeded 80%, demonstrating a non-cytotoxic profile, even after extended exposure.

Comparing the values obtained for low concertation of glyphosate and Roundup^®^, it can be observed that pure glyphosate exhibits a greater adverse effect. These results seem to be unexpected, but can be attributed to the fact that the commercial formulation contains other substances and, very importantly, contains glyphosate in the form of isopropylamine salt. For a proper comparation, and to elucidate this aspect, in the future tests including isopropylamine salts of glyphosate will be considered.

Levene’s Test was used for homogeneity of variance. The median-based test suggests that the variances were homogeneous (*p* = 0.994). A three-way ANOVA was used to evaluate the effects of compounds, concentration, and exposure time on activity. A statistically significant interaction between substance and concentration was observed (F(4, 60) = 53.065, *p* < 0.001), indicating that the relative performance of the two compounds varied with concentration. There was also a significant interaction between concentration and day (F(8, 60) = 2.812, *p* = 0.010). The main effect of substance was not statistically significant (F(1, 60) = 3.729, *p* = 0.058); the significant interaction between substance and concentration makes this main effect less relevant, as substances behave differently at different concentrations.

A post hoc Tukey analysis showed that the lowest concentrations (1 μM, 10 μM, and 250 μM) did not differ significantly. However, significant differences emerged at higher concentrations. The 500 μM concentration differed significantly from all lower doses (*p* < 0.001), and the highest concentration, 1 mM, differed significantly from all other concentrations tested (*p* < 0.001). These results are presented in Table 8.

**Figure 9:**
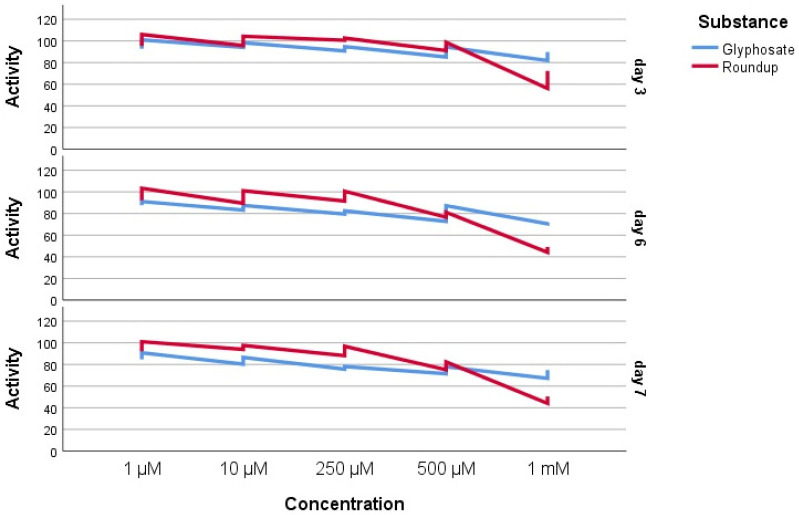
Multiple lines of results by concentration of glyphosate and Roundup formulation (created with SPSS).

The data in Figure 9 show a clear dose-dependent interaction.

## 4. Discussion

The world’s leading pesticides are glyphosate-based herbicides (GBHs), and their toxicity is highly debated [48]. Biomonitoring of glyphosate is very important given its persistence in the environment and possible adverse effects on non-target organisms, although glyphosate exhibits lower bioaccumulation than its formulations [48].

Glyphosate is currently the most widely used herbicide in the world. Although it is considered a safe herbicide, its excessive use generates chronic effects on the environment and on humans. The specialised literature has shown that glyphosate is a carcinogen and can cause organ failure by inhibiting acetylcholinesterase and inducing oxidative stress in non-mammalian species. In addition, the International Agency for Research on Cancer (IARC) has classified glyphosate in “Category 2a”, probably carcinogenic to humans [49,50].

Glyphosate was found in a few water samples, with concentrations ranging from near the limit of quantification to approximately 9.6 µg/mL. A substantial number of samples had absorbance values below the limit of detection, indicating either the absence or negligible presence of glyphosate at those locations. Many of the samples analysed were not within the concentration range that allows for accurate and precise determination of these values. Five commercially bottled drinking water samples were analysed. Glyphosate concentrations were either not detected or below the method’s limit of quantification (Table 5), with values around 1.14 µg/mL.

Glyphosate concentrations measured in soil samples showed substantial variability, ranging from values near the method’s LOQ to levels exceeding 80 µg/mL, depending on the sampling location. Extraction controls (blank soil extracts) showed measurable background absorbance, and blank-corrected concentrations were used for quantification within each analytical batch. The observed variability may reflect differences in local agricultural practices, herbicide application patterns, and soil characteristics specific to the areas investigated.

A variety of glyphosate concentrations were detected in groundwater, surface waters, and soil samples. Groundwater has often served as the primary source of potable water supply. Numerous findings indicate that water sources in regions with extensive agricultural activity may be at significant risk of glyphosate contamination. The presence of glyphosate in water has been examined by several researchers, with findings summarised in Table 9.

The reaction with ninhydrin and the formation of Ruhemann’s Purple are common mechanisms for a wide range of compounds containing -NH_2_ groups. Concentrations (µg/L) were calculated from absorbance measurements at λ = 567 nm using an external calibration. Given the nonspecific nature of the colourimetric reaction and possible interference from structurally similar compounds, results are reported as apparent glyphosate concentrations. Although calibration was performed using analytical-grade glyphosate standards, environmental compounds may also react; therefore, confirmatory methods using chromatographic techniques coupled with mass spectrometric detection are required to provide improved selectivity and confirmation.

Although the ninhydrin reaction is not absolutely specific for glyphosate, the potential interference from naturally occurring amino acids and amines in environmental matrices is limited because their concentrations are substantially lower than the method’s working range [59].

The choice of a spectrophotometric method as the main quantification approach, with UHPLC-MS/MS used as a confirmatory technique, was aligned with the objective of the environmental monitoring study.

In regions with limited analytical infrastructure, such as northeastern Romania, access to state-of-the-art instruments, including UHPLC-MS/MS, is often restricted by high acquisition and operating costs. In this context, the use of a cheap, accessible spectrophotometric method is a favourable solution for routine screening and monitoring.

This methodological choice enables analysis applicable in the field and in resource-limited contexts while maintaining analytical reliability through high-resolution confirmatory techniques.

The use of UHPLC-MS/MS for confirmatory purposes ensures specificity and analytical accuracy. This approach can be considered a current trend in environmental monitoring and is also described in other works as a cost-effective screening tool to prioritise sample analysis [60].

The proposed spectrophotometric method is rapid and cost-effective, and is very useful for preliminary monitoring of glyphosate in water and soil samples. The complementary UHRMS method confirms the glyphosate analysis in the samples studied by the spectrophotometric method.

The confirmation was assessed based on accurate mass, retention time, and MS/MS fragmentation. The identified fragments are similar to the literature data for glyphosate (79, 63 at 40 CE), supporting the validity of the analytical method. Thus, the UHPLC–UHRMS/MS method is suitable for the confirmatory identification of glyphosate in biological or environmental samples and can be subsequently integrated into a complete quantification and validation protocol. Given glyphosate’s high polarity, the lack of strong chromophores, and the high potential for matrix interferences, high-resolution mass spectrometry is an appropriate complementary approach for confirming the analyte’s identity.

Combining a simple, biomonitoring-friendly spectrophotometric method with an advanced confirmatory technique based on high-resolution mass spectrometry offers cost-effectiveness, sensitivity, and analytical specificity, which can be particularly useful for environmental studies, where numerous samples are analysed. Matrix diversity varies and can pose certain problems.

Although UHPLC-UHRMS/MS offers high specificity and structural certainty, its use in this study was limited by its technical complexity and associated costs. Compared to spectrophotometric methods, UHRMS analysis requires expensive, specialised equipment, highly qualified personnel, and longer analysis times, limiting its applicability. At the same time, the timing of the two methods differed, and the samples could not be analysed because the UHRMS method was developed later for this purpose.

In addition, glyphosate’s extreme polarity and its relatively low retention even on dedicated columns, such as Hypercarb, may increase susceptibility to matrix effects, especially in complex soil samples. For this reason, the UHRMS method was not used for quantification for the analysed samples, but was developed to be used in addition to the validated spectrophotometric method for biomonitoring.

In our opinion, the UHRMS method can serve as a complementary tool for confirmation rather than as an alternative to a spectrophotometric method (simpler, more accessible) for monitoring glyphosate in the environment.

The environmental footprint of modern agriculture is heavily influenced by the widespread use of plant protection products, which frequently raise concerns regarding bioaccumulation and persistent environmental toxicity [61,62]. To mitigate these risks, researchers are focusing on both minimizing initial application volumes and developing robust remediation technologies. One promising strategy involves the use of nano-herbicides, which permit significant reductions in the required application dosage—sometimes by up to 60%—while maintaining weed control efficacy, thereby lowering the total environmental burden [63]. Complementing this, Integrated Weed Management (IWM) strategies—such as diversified crop rotation, delayed drilling, and inversion ploughing—offer sustainable alternatives that reduce chemical dependence [35].

When contamination does occur, innovative remediation methods are critical. For soil decontamination, techniques such as biochar-based biofilters, mycoremediation using fungal strains like *Aspergillus oryzae*, and phytoremediation have proven effective in sequestering or breaking down residues [64,65,66]. Similarly, in water treatment, advanced oxidation processes (AOPs)—such as peracetic acid-assisted oxidation—and novel adsorbents, including metal–organic frameworks (MOFs), are being refined to selectively remove pollutants like glyphosate from aquatic systems. While the pursuit of safer alternative herbicides is ongoing, the inherent risks associated with bioaccumulation mean that the ultimate objective is often the transition toward holistic, non-chemical weed management systems [67,68].

Despite the extensive global use of glyphosate and numerous studies investigating its environmental occurrence, data regarding its presence in environmental matrices from Eastern Europe, particularly north-eastern Romania, remain limited. In addition, few studies have combined environmental monitoring with biological evaluation in human-derived cell models. This study addresses these gaps by providing new data on glyphosate contamination in soil and water samples from a previously under-investigated region and by evaluating its cytotoxic effects using primary human gingival fibroblast (hGF) cell lines.

The MTT reagent is used to study cell viability because it is reduced to formazan by metabolically active cells: it passes through the cell membrane, as well as the inner mitochondrial membrane of viable cells, due to its positive charge, as well as its lipophilic structure [69]. According to ISO 10993-5, cell viability values above 80% are considered non-cytotoxic; 60–80% weakly cytotoxic; 40–60% moderately cytotoxic; and below 40% strongly cytotoxic [50,70]. Reduction of MTT results in the disruption of the core tetrazole ring and the formation of a violet-blue, water-insoluble molecule called formazan, which provides the basis for the colourimetric measurement used in the MTT assay [69,71].

Cell viability for the active ingredient glyphosate (G) exceeded 80% at 1 μM and 10 μM across all exposure durations, indicating non-cytotoxicity (Table 7, Figure 6). At intermediate dosages (250 μM and 500 μM), both substances induced a mild cytotoxic response, with similar viability values recorded at 3, 6, and 7 days. Exposure to the maximum measured concentration (1 mM) induced mild cytotoxicity for both active components, with a slightly higher decrease in viability observed for glyphosate.

The results obtained on the effects of glyphosate and the commercial formulation on human gingival fibroblasts provide preliminary information on cell viability. A gradual decrease in cell viability was described following exposure to glyphosate alone, with values decreasing from 90.97% at 1 μM to 75.92% at 1 mM, suggesting a concentration-dependent trend. In contrast, the commercial formulation (Roundup) showed minimal impact at lower concentrations, followed by a marked reduction in viability at 1 mM (53.51%), indicating a potentially enhanced cytotoxic effect at higher exposure levels. At low and intermediate concentrations, cell viability was higher after Roundup treatment than in the glyphosate-alone group. This result may suggest a complex interaction between glyphosate and formulation co-formulants, such as surfactants, which may influence membrane permeability and cellular responses. The current in vitro cytotoxicity data for the active ingredient and its commercial formulation align with previously published research. Glyphosate-based herbicides, compared to glyphosate alone, can cause greater cell death and more significant genotoxic effects due to the presence of co-formulants [48]. The results indicate a relatively mild cytotoxicity profile for both glyphosate and its commercial formulation, especially at higher doses and prolonged exposure times. In vitro cytotoxicity studies of glyphosate and its formulation, compared to glycine, demonstrated dose-related cytotoxic effects of glyphosate and its Roundup Bioflow formulation, contradicting the lack of cellular toxicity observed for glycine [72]. The toxicity of glyphosate and its commercial formulations exhibits cytotoxic effects with significant dose-related differences in human and murine cell lines [72]. Also, the methodological variability inherent in in vitro cell analyses may contribute to this effect. Due to limited biological replication, the statistical results provide a solid basis for interpretation, but their confirmation in future studies with an expanded number of replicates is recommended.

To the best of our knowledge, no previous studies have specifically investigated the cytotoxic effects of glyphosate or glyphosate-based commercial formulations on primary human gingival fibroblasts (hGFs).

## 5. Conclusions

In conclusion, developing methods to monitor glyphosate residues and understanding their environmental impact is crucial. The association of a spectrophotometric method with a confirmatory UHPLC-UHRMS/MS technique constitutes an analytical strategy that enables the biomonitoring of glyphosate at the required specificity. This study supports the development of methods for monitoring glyphosate in the environment. Future research aims to expand large-scale, geographically extended monitoring of glyphosate in the environment, as well as other compounds used in agriculture and their metabolites, to provide a clearer assessment of environmental contamination and associated health risks. The only water sample with a measurable concentration (9.59 mg L^−1^) exceeds the environmental levels reported in consulted references (typically ≤204 µg L^−1^). The obtained soil apparent concentrations exceed the reported range of glyphosate concentrations in soil (0.0096–40.6 mg kg^−1^), suggesting either a possible contamination or a limitation in the specificity of the spectrophotometric method used. Despite the limitations of selectivity typical of spectrophotometric tests and the lower molecular specificity compared to chromatographic techniques, the simplicity of the procedure and the fact that it is accessible, rapid, and low-cost support its applicability as a practical screening method for monitoring glyphosate in environmental samples, such as water and soil. This study supports the development of methods for monitoring glyphosate in the environment. Future research aims to expand large-scale, geographically extended monitoring of glyphosate in the environment, as well as other compounds used in agriculture and their metabolites, to provide a clearer assessment of environmental contamination and associated health risks. Also, developing a UHPLC-MS/MS method for the quantification of polar pesticides (glyphosate, glufosinate, and fosetyl) will be useful for monitoring these compounds in environmental and biological samples and for correlating them with potential toxic effects on the human body. In addition, to our knowledge, the characterisation of the toxicity profiles of glyphosate and a glyphosate-based formulation in primary human gingival fibroblast (hGF) cell lines is addressed for the first time, with the commercial product Roundup showing cytotoxicity similar to that of the active compound at low and intermediate doses. Given the limited biological replication, these results should be interpreted as preliminary evidence. Future studies including larger sample sizes, additional cellular models, and mechanistic evaluation criteria are warranted to confirm and extend our findings. This study, by combining environmental monitoring with in vitro toxicological assessment on a relevant human cell model, provides a more comprehensive assessment of glyphosate exposure in NE Romania and the potential health risks.

## Figures and Tables

**Table 1:** Linearity and intra-assay precision of the spectrophotometric method for glyphosate determination.

Spiked Glyphosate Final Amount (μg/mL)	Set 1			Set 2			Set 3			Set 4			Set 5			CV%
	Absorbance	Back-Calculated Values	Accuracy	Absorbance	Back-Calculated Values	Accuracy	Absorbance	Back-Calculated Values	Accuracy	Absorbance	Back-Calculated Values	Accuracy	Absorbance	Back-Calculated Values	Accuracy	
1.055	0.02	1.302	123.4	0.02	1.488	141.0	0.01	1.233	116.8	0.02	1.579	149.7	0.02	1.594	151.1	24.8
1.319	0.02	1.302	98.7	0.02	1.488	112.8	0.02	1.814	137.5	0.01	0.970	73.5	0.02	1.594	120.8	24.8
1.649	0.03	1.893	114.8	0.03	2.098	127.2	0.02	1.814	110.0	0.02	1.579	95.8	0.03	2.219	134.6	21.1
2.061	0.04	2.485	120.6	0.03	2.098	101.8	0.02	1.814	88.0	0.03	2.189	106.2	0.03	2.219	107.7	23.6
2.57	0.04	2.485	96.7	0.04	2.707	105.3	0.03	2.395	93.2	0.03	2.189	85.2	0.03	2.219	86.3	16.1
3.221	0.05	3.077	95.5	0.04	2.707	84.1	0.04	2.977	92.4	0.05	3.409	105.8	0.04	2.844	88.3	12.4
4.026	0.07	4.260	105.8	0.07	4.537	112.7	0.06	4.140	102.8	0.07	4.628	115.0	0.06	4.094	101.7	8.3
5.033	0.09	5.444	108.2	0.08	5.146	102.3	0.09	5.884	116.9	0.07	4.628	92.0	0.07	4.719	93.8	12.5
6.291	0.1	6.036	95.9	0.09	5.756	91.5	0.1	6.465	102.8	0.11	7.067	112.3	0.1	6.594	104.8	7.1
7.864	0.13	7.811	99.3	0.13	8.195	104.2	0.12	7.628	97.0	0.11	7.067	89.9	0.12	7.844	99.7	6.9
9.83	0.15	8.994	91.5	0.17	10.634	108.2	0.16	9.953	101.3	0.15	9.506	96.7	0.15	9.719	98.9	5.7
12.88	0.2	11.953	92.8	0.17	10.634	82.6	0.19	11.698	90.8	0.2	12.555	97.5	0.19	12.219	94.9	6.4
15.36	0.25	14.911	97.1	0.24	14.902	97.0	0.24	14.605	95.1	0.24	14.994	97.6	0.23	14.719	95.8	2.9
19.2	0.34	20.237	105.4	0.3	18.561	96.7	0.31	18.674	97.3	0.31	19.262	100.3	0.3	19.094	99.4	5.3
24	0.4	23.787	99.1	0.4	24.659	102.7	0.4	23.907	99.6	0.4	24.750	103.1	0.39	24.719	103.0	1.1
30	0.51	30.296	101.0	0.5	30.756	102.5	0.52	30.884	102.9	0.48	29.628	98.8	0.48	30.344	101.1	3.6
Regression equation	y = 0.0169x − 0.002 R^2^ = 0.9969			y = 0.0164x − 0.0044 R^2^ = 0.9927			y = 0.0171x − 0.0112 R^2^ = 0.9963			y = 0.0164x − 0.0059 R^2^ = 0.9971			y = 0.016x − 0.0055 R^2^ = 0.9977			

**Table 2:** Linearity and intra-assay precision of the spectrophotometric method for glyphosate determination.

Parameter	Value
Coefficient of determination (R^2^)	0.9981
Slope	0.0166
Intercept	−0.0058

**Table 3:** Inter-assay precision of the spectrophotometric method for glyphosate determination.

Spiked Glyphosate Final Amount (μg/mL)	Set 1			Set 2			Set 3			Set 4			Set 5			Set 6			CV%
	Absorbance	Back-Calculated Values	Accuracy	Absorbance	Back-Calculated Values	Accuracy	Absorbance	Back-Calculated Values	Accuracy	Absorbance	Back-Calculated Values	Accuracy	Absorbance	Back-Calculated Values	Accuracy	Absorbance	Back-Calculated Values	Accuracy	
1.055	0.01	1.234	116.9	0.04	2.000	189.5	0.02	1.224	116.0	0.01	1.373	130.1	0.03	1.740	164.9	0.03	1.706	161.7	51.9
1.319	0.01	1.234	93.5	0.03	1.479	112.1	0.03	1.745	132.3	0.02	1.891	143.4	0.03	1.740	131.9	0.02	1.171	88.8	35.0
1.649	0.02	1.722	104.4	0.04	2.000	121.2	0.05	2.786	169.0	0.02	1.891	114.7	0.04	2.260	137.1	0.03	1.706	103.4	36.3
2.061	0.02	1.722	83.5	0.04	2.000	97.0	0.04	2.266	109.9	0.02	1.891	91.8	0.03	1.740	84.4	0.03	1.706	82.8	29.8
2.57	0.05	3.185	123.9	0.04	2.000	77.8	0.04	2.266	88.2	0.04	2.927	113.9	0.04	2.260	88.0	0.04	2.241	87.2	9.8
3.221	0.06	3.673	114.0	0.07	3.563	110.6	0.05	2.786	86.5	0.04	2.927	90.9	0.06	3.302	102.5	0.06	3.310	102.8	18.2
4.026	0.07	4.161	103.3	0.1	5.125	127.2	0.06	3.307	82.1	0.06	3.964	98.5	0.08	4.344	107.9	0.08	4.380	108.8	20.2
5.033	0.08	4.649	92.3	0.1	5.125	101.8	0.1	5.391	107.1	0.08	5.000	99.3	0.09	4.865	96.7	0.09	4.914	97.6	9.9
6.291	0.11	6.112	97.1	0.12	6.167	98.0	0.1	5.391	85.7	0.09	5.518	87.7	0.1	5.385	85.6	0.13	7.053	112.1	13.6
7.864	0.14	7.576	96.3	0.15	7.729	98.2	0.15	7.995	101.7	0.16	9.145	116.3	0.14	7.469	95.0	0.14	7.588	96.5	5.6
9.83	0.18	9.527	96.9	0.17	8.771	89.2	0.15	7.995	81.3	0.16	9.145	93.0	0.16	8.510	86.6	0.18	9.727	99.0	7.3
12.88	0.23	11.966	92.9	0.22	11.375	88.3	0.21	11.120	86.3	0.2	11.218	87.1	0.23	12.156	94.4	0.21	11.332	88.0	5.6
15.36	0.3	15.380	100.1	0.29	15.021	97.7	0.29	15.286	99.5	0.28	15.363	100.0	0.3	15.802	102.9	0.28	15.075	98.1	3.1
19.2	0.39	19.771	102.9	0.36	18.667	97.2	0.36	18.932	98.6	0.36	19.508	101.6	0.39	20.490	106.7	0.38	20.422	106.4	4.0
24	0.49	24.649	102.7	0.47	24.396	101.6	0.44	23.099	96.2	0.45	24.171	100.7	0.48	25.177	104.9	0.46	24.701	102.9	4.0
30	0.59	29.527	98.4	0.6	31.167	103.8	0.56	29.349	97.8	0.57	30.389	101.3	0.55	28.823	96.1	0.55	29.513	98.4	3.7
Regression equation	y = 0.0205x − 0.0153, R^2^ = 0.9975			y = 0.0192x + 0.0016 R^2^ = 0.9932			y = 0.0185x − 0.0035 R^2^ = 0.993			y = 0.0193x − 0.0165 R^2^ = 0.9945			y = 0.0192x − 0.0034 R^2^ = 0.9922			y = 0.0187x − 0.0019 R^2^ = 0.9948			

Data are expressed as mean absorbance ± SD (*n* = 6). Higher CV values observed at the lowest concentrations are expected because the measurements are close to the quantification limit.

**Table 4:** Linearity and intra-assay precision of the spectrophotometric method for glyphosate determination.

Parameter	Value
Number of samples	40
Samples below LOQ	39
Samples with detectable glyphosate	1
Maximum concentration (µg/mL)	9.59

**Table 5:** Glyphosate determination in bottled water samples.

Sample ID	Absorbance (567 nm)	Glyphosate Apparent Concentration (µg/mL)
Water 1	0.00	0.57
Water 2	0.01	1.14
Water 3	0.01	1.14
Water 4	0.01	1.14
Water 5	0.01	1.14

**Table 6:** Glyphosate apparent concentrations determined in soil samples (spectrophotometric method, 567 nm).

Analytical Batch	Sample ID	Absorbance (567 nm)	Glyphosate Apparent Concentration (µg/mL)	Glyphosate Apparent Concentration (µg/g)
Set 1	Blank soil extract	0.10	6.38	15.950
Set 1	P1	0.54	35.33	88.325
Set 1	P2	0.81	53.09	132.725
Set 1	P3	0.19	12.30	30.750
Set 1	P4	0.08	5.07	12.675
Set 1	P5	0.26	16.91	42.275
Set 1	P6	1.24	81.38	203.450
Set 1	P7	0.29	18.88	47.200
Set 1	P8	0.39	25.46	63.650
Set 1	P9	0.12	7.70	19.250
Set 2	Blank soil extract	0.11	6.29	15.725
Set 2	P10	0.46	25.63	64.075
Set 2	P11	0.05	2.98	7.450
Set 2	P12	0.11	6.29	15.725
Set 2	P13	0.23	12.92	32.300
Set 2	P14	0.10	5.74	14.350
Set 2	P15	0.12	6.85	17.125
Set 2	P16	0.08	4.64	11.600
Set 2	P17	0.02	1.32	3.300
Set 2	P18	0.29	16.24	40.600
Set 2	P19	0.08	4.76	11.900
Set 2	P20	0.41	23.09	57.725
Set 2	P21	0.26	14.76	36.900
Set 2	P22	0.14	8.09	20.225
Set 2	P23	0.22	12.53	31.325
Set 2	P24	0.05	3.09	7.725
Set 2	P25	0.07	4.20	10.500
Set 2	P26	0.07	4.20	10.500
Set 2	P27	0.05	3.09	7.725
Set 2	P28	0.15	8.64	21.600

“Analytical batch” indicates measurements performed using freshly prepared calibration curves on different analytical runs; blank soil extracts are reported for the corresponding batches.

**Table 7:** Cell viability (%) of hGF after exposure to glyphosate and the commercial formulation Roundup^®^.

Substance	Exposure Time (Days)	Control	1 µM	10 µM	250 µM	500 µM	1 mM
Glyphosate	3	100 ± 5.93	96.15 ± 4.31	95.82 ± 2.25	92.85 ± 1.99	90.54 ± 5.81	86.36 ± 4.07
	6	100 ± 6.79	89.63 ± 1.76	85.10 ± 2.17	81.78 ± 7.9	81.10 ± 1.49	70.98 ± 0.72
	7	100 ± 4.04	87.12 ± 3.19	83.90 ± 3.24	77.26 ± 1.39	75.05 ± 3.3	70.42 ± 3.96
Roundup^®^	3	100 ± 5.93	100.2 ± 5.33	100.9 ± 4.62	101.8 ± 1.06	95.60 ± 3.4	66.23 ± 8.82
	6	100 ± 6.79	96.50 ± 5.95	95.14 ± 5.78	96.20 ± 4.54	78.68 ± 2.28	47.42 ± 2.81
	7	100 ± 4.04	96.18 ± 4.48	95.84 ± 1.81	93.49 ± 4.69	77.93 ± 3.84	46.88 ± 3.29

**Table 8:** Effects of glyphosate and Roundup at different concentrations.

Concentration	Glyphosate (Mean ± SD)	Roundup (Mean ± SD)	*p*-Value
1 μM	90.97 ± 4.92	97.63 ± 4.98	0.011 *
10 μM	88.27 ± 6.11	97.33 ± 4.71	0.003 *
250 μM	83.74 ± 7.18	97.19 ± 4.96	<0.001 *
500 μM	82.46 ± 8.33	84.07 ± 9.16	0.700
1 mM	75.92 ± 8.34	53.51 ± 10.73	<0.001 *

Statistical significance was determined using Independent-Sample *t*-Tests at each concentration. (*) indicates a statistically significant difference between substances (*p* < 0.05).

**Table 9:** Glyphosate concentrations in environmental matrices (soil, groundwater, and surface water) reported in different countries.

Country	Sample Type	Glyphosate Concentration	References
Greece	soil	0.026–40.6 μg g^−1^	[51]
Finland	soil	0.1 mg kg^−1^–3 mg kg^−1^	[52]
Canada	soil	maximum 0.47 mg·kg^−1^	[53]
China	soil	888.85 µg kg^−1^	[27]
U.S.	soil	median: 9.6 µg kg^−1^	[24]
Portugal	groundwater	maximum 4.69 µg L^−1^	[54]
U.S.	groundwater	up to 2 µg L^−1^	[24]
Brazil	tap water	0.015 mg L^−1^–0.18 mg L^−1^	[55]
U.S.	surface water	0.03 µg L^−1^	[24]
Italy	surface water	0.1 µg L^−1^–108 µg L^−1^	[56]
Brazil	surface water	24 mg L^−1^–6.1 mg L^−1^	[57]
Switzerland	surface water	0.11 µg L^−1^–2.1 µg L^−1^	[58]
China	surface water	0–204.0 µg L^−1^	[27]

## Data Availability

The original contributions presented in this study are included in the article. Further inquiries can be directed to the corresponding author.
